# Assessment of the Position of Maxillary Sinus Floor in Relation to Alveolar Ridge, Location of Sinus Septa, and Posterior Superior Alveolar Artery in the Chhattisgarh Population: A Cone-Beam Computed Tomography Analysis

**DOI:** 10.7759/cureus.113084

**Published:** 2026-07-21

**Authors:** Avinash Kumar, Saumya Sharma, Sanjeev Singh, Priyabrata Jena, Baby Jaman, Aviraj Gill, Pankaj Bajaj

**Affiliations:** 1 Department of Prosthodontics, Maitri College of Dentistry and Research Centre, Durg, IND; 2 Department of Prosthodontics, Triveni Institute of Dental Sciences Hospital and Research Centre, Bilaspur, IND

**Keywords:** alveolar ridge, cbct, dental implants, maxillary sinus, posterior superior alveolar artery, sinus septa

## Abstract

Background: Substantial anatomical diversity exists within the maxillary sinus, particularly in relation to the alveolar crest, sinus septa, and the posterior superior alveolar artery (PSAA). Such variations hold important clinical implications, especially in procedures involving implant placement and sinus floor elevation.

Objective: This study aimed to evaluate the positional relationship between the maxillary sinus floor and the alveolar ridge, determine the prevalence and distribution of maxillary sinus septa, and assess the anatomical course of the PSAA in a Chhattisgarh population using cone-beam computed tomography (CBCT).

Materials and methods: A prospective observational design with a cross-sectional approach was adopted. The sample comprised 120 CBCT datasets from individuals aged 30-65 years. Three-dimensional reconstructed images were evaluated to determine the vertical separation between the sinus floor and the alveolar crest in the region of the first molar. Identified sinus septa were grouped according to their position into anterior, middle, or posterior categories. The trajectory of the PSAA was assessed and classified into intraosseous, intrasinus, or superficial patterns. Statistical procedures were applied to explore potential relationships with patient gender and dentition status.

Results: In most evaluated cases, sinus septa were not observed (right side: 69.2%; left side: 70.0%). When present, septa showed a higher frequency in the anterior segment. The PSAA most commonly followed an intraosseous pathway on both sides (right: 55.8%; left: 53.3%), with intrasinus and superficial courses occurring less frequently. The measured vertical distance between the sinus floor and the alveolar crest showed no statistically significant variation when stratified by gender or dentition status (p > 0.05). Additionally, no meaningful correlations were identified between anatomical features and the studied variables.

Conclusion: Anatomical variations of the maxillary sinus and PSAA are common but do not appear to be significantly influenced by gender or dentition status. Preoperative CBCT evaluation remains essential for minimizing surgical risks and optimizing treatment outcomes in the posterior maxilla.

## Introduction

The maxillary sinus represents the largest air-filled cavity among the paranasal sinuses and is located within the maxillary bone, typically exhibiting a pyramidal configuration. Considerable variation can occur in its size, morphology, and spatial orientation, both across individuals and between the two sides in the same individual. These differences may be influenced by demographic and biological factors, including age, sex, and ethnic background. The sinus floor maintains a close anatomical association with the roots of posterior maxillary teeth and may extend between adjacent roots, occasionally allowing root apices to project into the sinus space. This proximity contributes to the clinical relevance of the maxillary sinus in diagnostic evaluation, therapeutic planning, and surgical interventions in the posterior maxilla [1].

Implant-supported rehabilitation is frequently employed in cases of tooth loss in the posterior maxillary region. Nevertheless, treatment in this area can present challenges due to compromised bone density, ongoing resorption of the alveolar crest, and progressive sinus pneumatization following tooth extraction. Reduction in vertical bone dimension within this region may adversely affect the predictability of implant placement in the posterior maxilla. In addition, the close anatomical proximity between the sinus cavity and the alveolar crest increases susceptibility to procedural complications, including disruption of the Schneiderian membrane, inflammatory sequelae, and sinus-associated pathology during augmentation interventions. A significant proportion of sinus conditions are attributable to dental origins, reinforcing the importance of detailed assessment of the anatomical interface between the sinus and surrounding dentoalveolar structures [2].

Osseous septations within the maxillary sinus represent a well-documented anatomical variation and are frequently described as antral septa. Their presence was initially reported by Underwood in 1910, with emphasis on their occurrence and distribution patterns within the sinus cavity [3]. These bony projections may arise from the sinus floor or lateral walls and can partially or completely partition the sinus into discrete compartments. Based on developmental origin, they are classified as primary septa, formed during maxillary growth, or secondary septa, which develop irregular pneumatization of the sinus following tooth loss [4].

The reported frequency of sinus septa varies widely in the literature, with values ranging from approximately 16% to 58%, influenced by differences in study populations and diagnostic imaging techniques [5]. From a clinical standpoint, their presence is relevant due to the increased complexity they introduce during sinus augmentation procedures, particularly with regard to the elevated risk of membrane perforation. Furthermore, septa may alter the internal morphology of the sinus by forming recesses or compartments, thereby complicating surgical access and operative manipulation [5].

Both the posterior superior alveolar artery (PSAA) and the infraorbital artery originate as branches of the maxillary artery and contribute to the vascular supply of the lateral sinus wall and the Schneiderian membrane. The maxillary artery constitutes the principal source of perfusion for the sinus and its mucosal lining. This vascular arrangement was first described by Strong in 1934 [4].

Anastomotic connections between the posterior superior alveolar artery and the infraorbital artery may be identified in intraosseous as well as extraosseous locations. The intraosseous form, commonly referred to as the alveolar antral artery, provides vascular branches to the sinus membrane and adjacent periosteum. Extraosseous communications have also been documented in anatomical and cadaveric investigations, including those reported by Traxler [6].

Failure to identify anatomical variations of the maxilla prior to surgical intervention may increase the likelihood of intraoperative complications such as hemorrhage, neural damage, tissue trauma, and suboptimal outcomes in implant or regenerative procedures. Therefore, comprehensive preoperative imaging evaluation is essential. Cone-beam computed tomography (CBCT) provides high-resolution, three-dimensional visualization of maxillofacial structures, enabling precise identification of anatomical details that may not be evident on conventional two-dimensional radiographs while minimizing the effects of superimposition and imaging artifacts [7].

## Materials and methods

Study design

A prospective cross-sectional observational study was conducted in a population from Chhattisgarh. The dataset comprised CBCT scans acquired between April 2024 and February 2026. These scans were originally obtained for preoperative assessment prior to implant placement or other regenerative interventions, including sinus floor elevation procedures. The imaging data were evaluated to determine the spatial relationship between the maxillary sinus floor and the alveolar crest, as well as to analyze the distribution of sinus septa and the anatomical pathway of the PSAA. Ethical clearance for the study was granted by the Institutional Ethics Committee of Maitri College of Dentistry and Research Centre, Durg, India (Approval No.: MCDRC/2024/APRIL/422; approved on April 2, 2024). Informed consent was obtained from all individuals whose imaging records were included in the analysis.

A total of 120 CBCT scans were analyzed, collected over a period of approximately 22 months (April 2024 to February 2026). The study sample included both partially and completely edentulous maxillae from male and female participants aged between 30 and 65 years.

Inclusion criteria comprised individuals within the specified age range who presented with either partial or complete edentulism of the maxilla. Only CBCT scans demonstrating clear bilateral visualization of the maxillary sinuses and absence of pathological findings were considered suitable for inclusion. These scans were further assessed for the presence of sinus septa and the course of the PSAA.

Exclusion criteria comprised any prior history of maxillofacial trauma or fractures, impacted or supernumerary teeth within the region of interest, previous surgical interventions involving the maxilla, systemic or neurological disorders, syndromic conditions, pregnancy, and extensive bony lesions. Additionally, scans exhibiting dental implants or imaging artifacts in the sinus region were omitted from the study.

Radiographic equipment and imaging protocol

All CBCT examinations were performed using a Genoray Papaya 3D Plus system (Genoray Co., Ltd., Seoul, South Korea) under standardized exposure parameters, including a tube voltage of 60-90 kVp and a tube current of 8-12 mA. The imaging field of view ranged from 16 × 8 cm to 10 × 10 cm, with a voxel size of 0.100-0.200 mm. Image reconstruction was carried out in cylindrical mode, and subsequent analysis was conducted using dedicated imaging software (Theia, Genoray) on a computer system equipped with an Intel i5 (12th generation) processor (Intel Corporation, Santa Clara, California).

All scans were obtained by a single experienced radiologist following consistent patient positioning and exposure protocols. The acquired images were stored in DICOM format, and post-processing adjustments, including brightness and contrast optimization, were applied when necessary to enhance image interpretation and measurement accuracy.

Image evaluation and measurement protocol

The CBCT datasets were examined using multiplanar reconstruction (MPR), incorporating axial, sagittal, and coronal views. Panoramic reconstructions were additionally generated at slice intervals of 1, 5, and 10 mm. Three-dimensional reconstructions were utilized to identify the trajectory of the PSAA, detect the presence and location of sinus septa, and assess the spatial relationship between the maxillary sinus floor and the alveolar crest. All measurements were conducted bilaterally to ensure a comprehensive evaluation.

**Figure 1:**
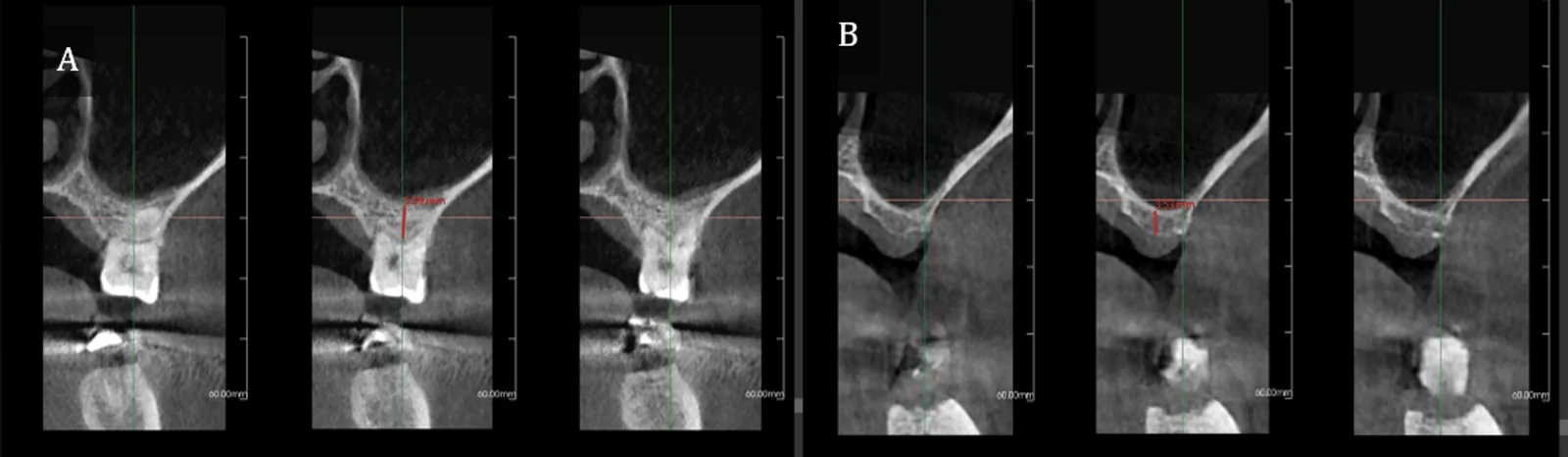
Position of maxillary sinus floor from the ridge. (A) Dentulous. (B) Edentulous. Representative CBCT cross-sectional images demonstrating the measurement of the vertical distance between the maxillary sinus floor and the alveolar ridge. (A) Dentulous maxilla, with the measurement taken from the sinus floor to the furcation of the first molar. (B) Edentulous maxilla, with the measurement taken from the sinus floor to the crest of the residual alveolar ridge. Red markings indicate the measured distance.

The principal measurement focused on evaluating the vertical separation between the alveolar crest and the maxillary sinus floor in the first molar region. In dentate cases, this measurement extended from the sinus floor to the level of molar furcation, whereas in edentulous cases, it was determined from the sinus floor to the crest of the residual alveolar ridge, as illustrated in Figure 1.

**Figure 2:**
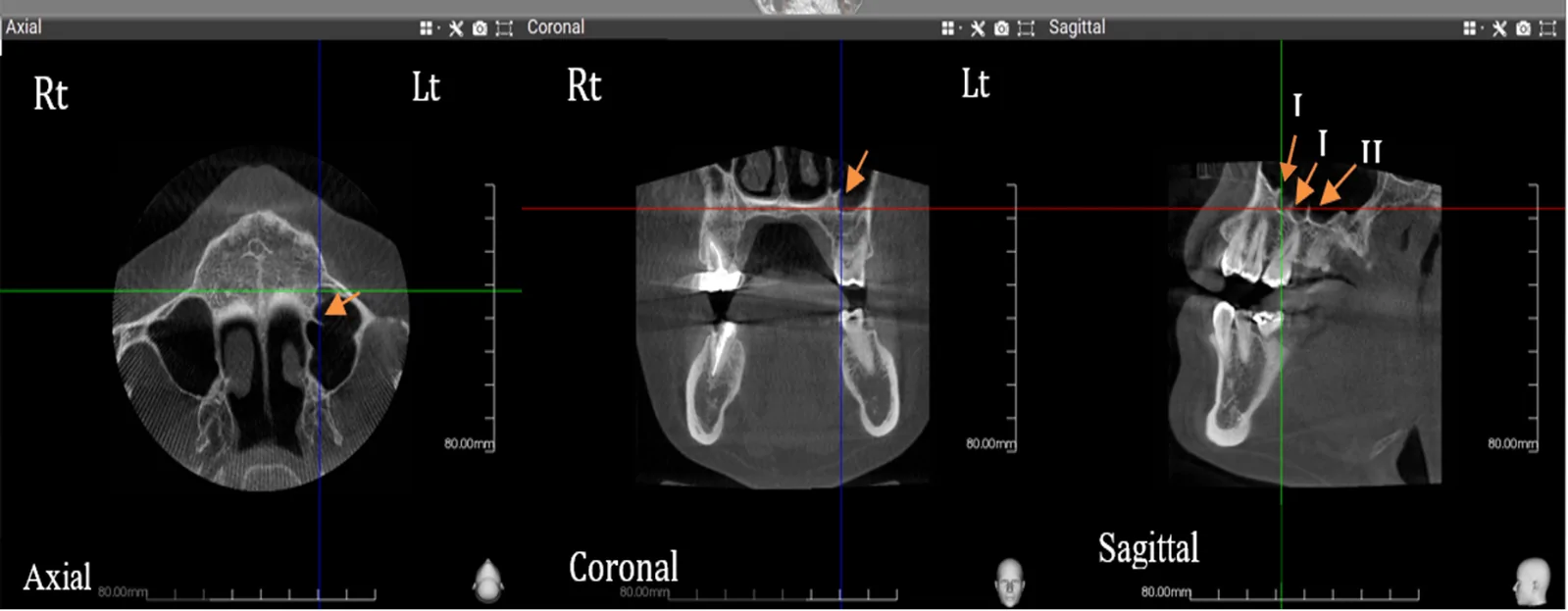
Positions of maxillary sinus septa in MPR (axial, coronal, and sagittal views) (I, Anterior; II, Middle; III, Posterior). Multiplanar reconstruction (MPR) CBCT images (axial, coronal, and sagittal views) demonstrating the location of maxillary sinus septa. Septa are classified based on their position as (I) anterior (mesial to the second premolar), (II) middle (between the second premolar and second molar), and (III) posterior (distal to the second molar). Arrows indicate the identified septa in each plane.

The presence of maxillary sinus septa was assessed and categorized according to their anatomical position. Septa located anterior to the second premolar were designated as anterior, those between the second premolar and second molar were classified as middle, and those situated posterior to the second molar were considered posterior (Figure 2).

All CBCT images were evaluated by a single examiner who underwent calibration prior to the initiation of the study to ensure consistency in image interpretation and measurements. Examiner calibration was performed using a subset of CBCT scans that were independently assessed on two separate occasions before the commencement of the final analysis.

To evaluate intraobserver reliability, 30 randomly selected CBCT scans were re-examined after an interval of two weeks under identical viewing conditions. The repeated measurements demonstrated excellent agreement and high reproducibility. As the measurements were performed by a single calibrated examiner, interobserver reliability analysis was not applicable in the present study. During image evaluation and data recording, the examiner was blinded to participant demographic information, including gender and dentition status, in order to minimize observer bias and improve methodological rigor. Intraclass correlation coefficient (ICC) analysis was used for continuous measurements of sinus floor-to-alveolar ridge distance, while Cohen's kappa statistics were applied for categorical assessments, including sinus septa location and PSAA classification.

**Figure 3:**
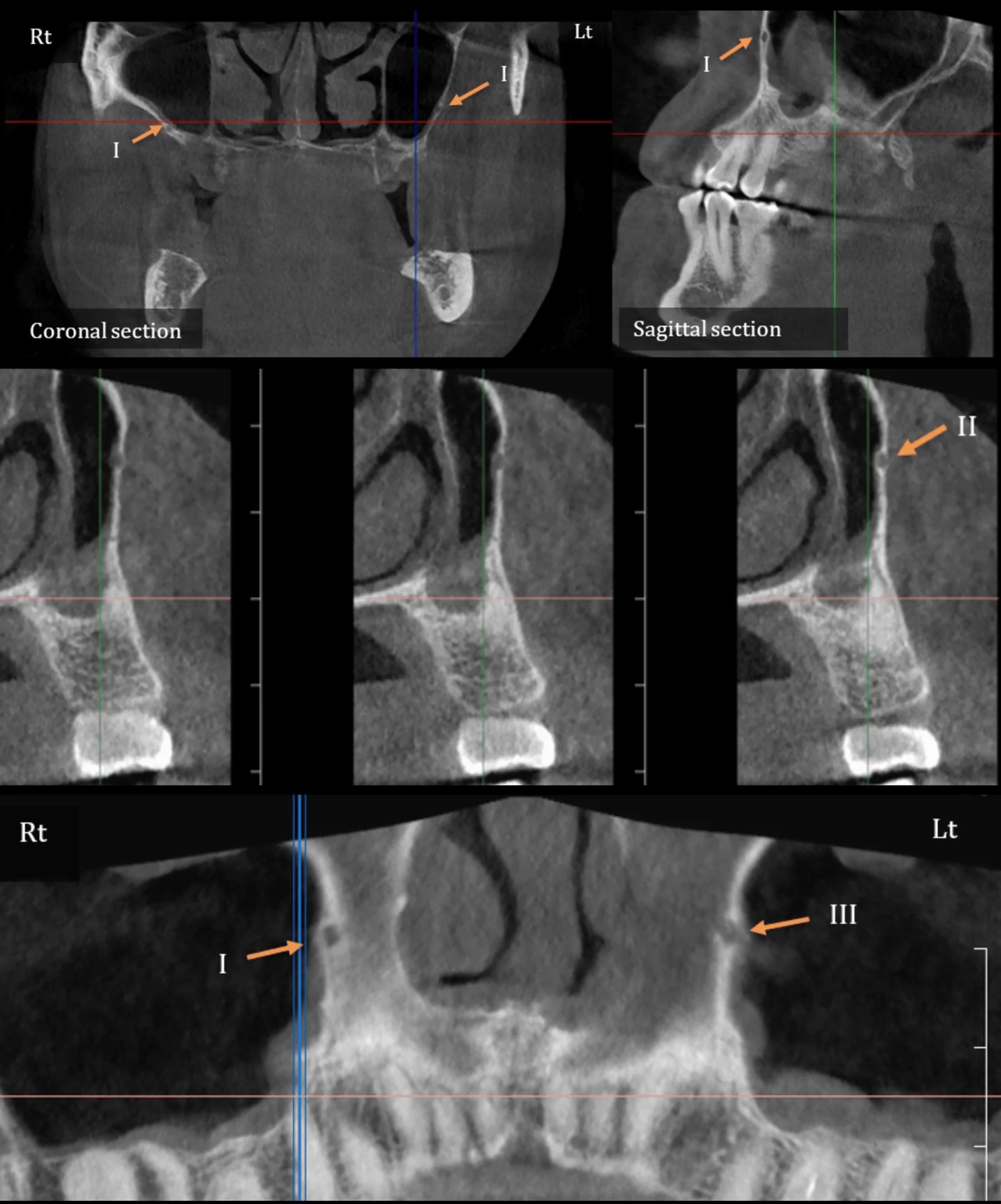
Different variations of the PSAA in coronal, sagittal, and curved sections (I, Intraosseous; II, Intrasinus; III, Superficial). CBCT images in coronal, sagittal, and curved sections demonstrating variations in the course of the posterior superior alveolar artery (PSAA). The PSAA is classified based on its relationship to the lateral wall of the maxillary sinus as (I) intraosseous, (II) intrasinus, and (III) superficial. Arrows indicate the location of the artery in each section.

Evaluation of the PSAA was performed using coronal, sagittal, and curved sectional CBCT images to assess anatomical variations. Based on its spatial relationship to the lateral wall of the maxillary sinus, the artery was classified into three patterns: intraosseous (Type I), intrasinus (Type II), and superficial (Type III), as demonstrated in Figure 3. 

Sample size calculation

The required sample size for this study was determined based on previously published CBCT investigations [7] evaluating maxillary sinus anatomy. Assuming an expected prevalence of sinus septa or PSAA variations between 40% and 60%, with a 95% confidence level and an allowable margin of error ranging from 8% to 10%, the calculated sample size was considered adequate for reliable statistical analysis. The estimation was performed using the standard formula for sample size determination 

$n=\frac{z^{2}\times p\times q}{d^{2}}$

where n represents the sample size, z corresponds to the standard normal deviate at a 95% confidence level (1.96), p denotes the expected prevalence, q is calculated as (1 − p), and d represents the permissible margin of error. Based on this calculation, the minimum required sample size ranged between 100 and 120 subjects.

Statistical analysis

Data analysis was conducted using IBM SPSS Statistics for Windows, Version 22 (released 2013; IBM Corp., Armonk, New York). Descriptive statistics were applied to summarize the dataset. Categorical variables, including sex, dentition status, location of sinus septa, and PSAA classification, were reported as frequencies and percentages. Continuous variables, such as the vertical distance between the maxillary sinus floor and the alveolar crest, were expressed as mean values along with standard deviations.

Inferential statistical methods were employed to evaluate relationships between categorical variables using the chi-square test. Differences in mean values between independent groups (e.g., male versus female participants and dentate versus edentulous subjects) were assessed using independent samples t-tests. A confidence level of 95% was adopted, and a p-value less than 0.05 was considered indicative of statistical significance. Intraobserver reliability was evaluated by reassessing 25% of the randomly selected CBCT scans. Cohen's kappa statistics were used for categorical variables, while intraclass correlation coefficients (ICC) were calculated for continuous measurements. Along with p-values, 95% confidence intervals and appropriate effect size measures were calculated to assess the precision and magnitude of observed differences between groups.

## Results

**Figure 4:**
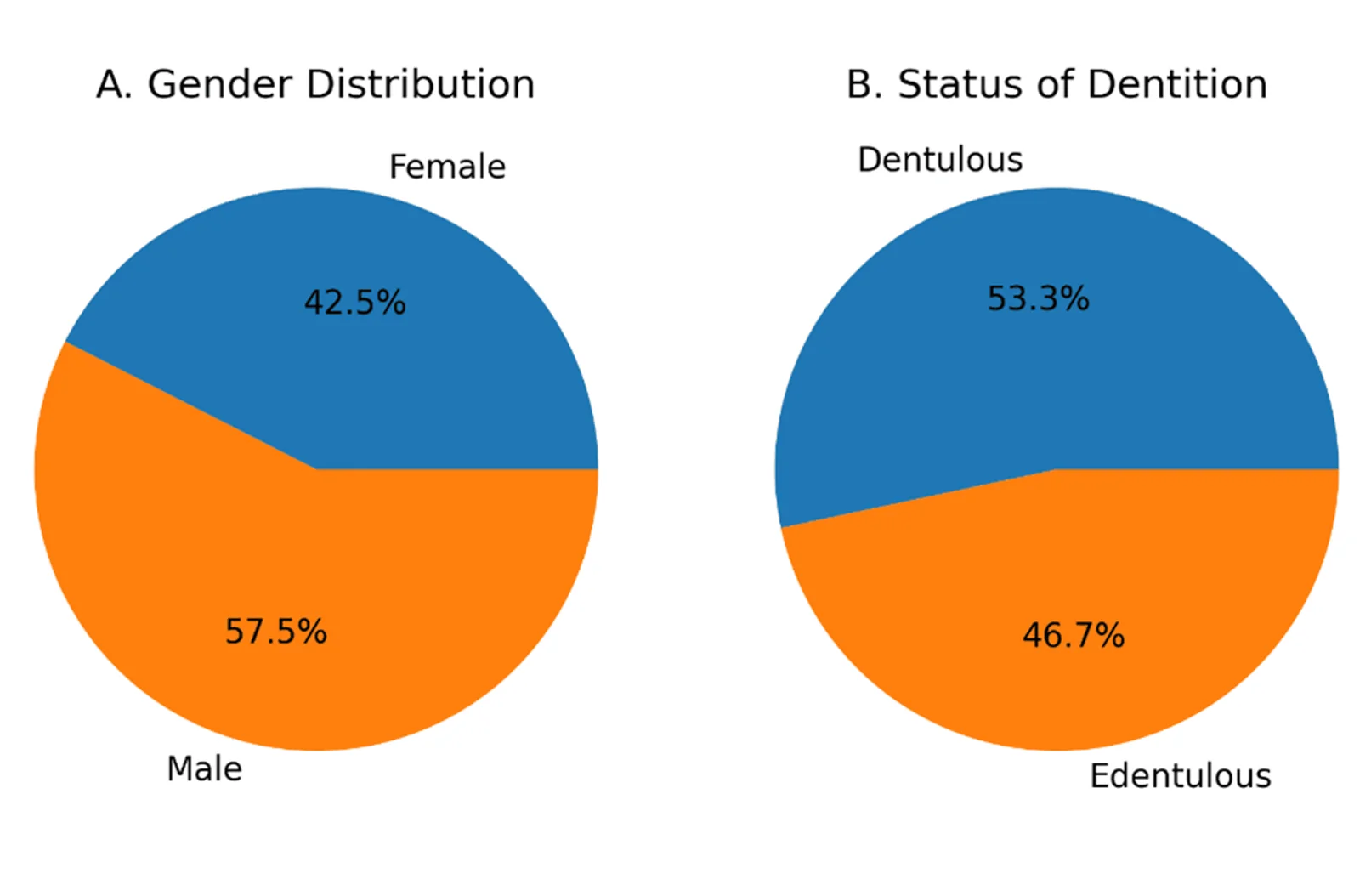
Distribution of study population according to gender and dentition status. Pie charts illustrating the demographic distribution of the study sample. (A) Gender distribution showing the proportion of males (57.5%) and females (42.5%). (B) Dentition status showing the proportion of dentulous (53.3%) and edentulous (46.7%) subjects.

The study sample consisted of 120 individuals, including 69 males (57.5%) and 51 females (42.5%). With respect to dentition status, 64 participants (53.3%) were dentate, whereas 56 (46.7%) were edentulous (Figure 4).

Evaluation of maxillary sinus septa revealed that septa were absent in the majority of cases on both sides. On the right side, septa were not identified in 83 cases (69.2%), while on the left side, absence was noted in 84 cases (70.0%). Among cases where septa were present, anterior septa were the most frequently observed (right: 17.4%; left: 13.4%), followed by middle (right: 6.7%; left: 8.3%) and posterior locations (right: 6.7%; left: 8.3%) as presented in Table 1.

**Table 1:** Distribution of maxillary sinus septa according to location on the right and left sides. Distribution of maxillary sinus septa based on their location on the right and left sides. The values are expressed as frequency (N) and percentage (%). Septa were categorized as anterior, middle, and posterior according to their anatomical position, while “absent” indicates cases with no septa detected.

Variables	Location of the right side maxillary sinus septa		Location of the left side maxillary sinus septa	
	N	%	N	%
Absent	83	69.2	84	70.0
Anterior	21	17.4	16	13.4
Middle	8	6.7	10	8.3
Posterior	8	6.7	10	8.3

Assessment of the PSAA demonstrated that the intraosseous pattern was the most prevalent on both sides. On the right side, 55.8% of cases exhibited an intraosseous course, followed by intrasinus (35.0%) and superficial (9.2%) patterns. Similarly, on the left side, the intraosseous type was most common (53.3%), with intrasinus (37.5%) and superficial (9.2%) patterns occurring less frequently, as presented in Table 2.

**Table 2:** Distribution of the PSAA according to location on the right and left sides. Distribution of the posterior superior alveolar artery (PSAA) based on its location on the right and left sides. The values are expressed as frequency (N) and percentage (%). The PSAA was classified according to its relation to the lateral wall of the maxillary sinus as intraosseous, intrasinus, and superficial.

Variables	Location of PSAA: right side		Location of PSAA: left side	
	N	%	N	%
Intraosseous	67	55.8	64	53.3
Intrasinus	42	35.0	45	37.5
Superficial	11	9.2	11	9.2

The mean distance between the maxillary sinus floor and the alveolar ridge at the first molar region was marginally higher in males than in females on both sides. On the right side, the mean distance was 7.47 ± 2.83 mm in males and 6.90 ± 3.04 mm in females (p = 0.29). On the left side, the corresponding values were 7.16 ± 2.74 mm and 7.00 ± 2.55 mm, respectively (p = 0.74). These differences were not statistically significant, as presented in Table 3.

**Table 3:** A comparison of mean distance between maxillary sinus floor and alveolar ridge according to gender. The table summarizes the mean distance (in mm) between the maxillary sinus floor and the alveolar ridge on the right and left sides in male and female subjects. Data are presented as mean ± standard deviation. The mean difference, t-value, and p-value were determined using an independent samples t-test to evaluate statistical significance between the groups.

Variables	Sex	N	Mean	Std. Deviation	Mean diff	T value	P value
Position of maxillary sinus right	Male	69	7.4738	2.83510	0.568	1.051	0.29
	Female	51	6.9055	3.04855			
Position of maxillary sinus left	Male	69	7.1661	2.74262	0.158	0.322	0.74
	Female	51	7.0076	2.55103			

Similarly, no statistically significant differences were found between dentulous and edentulous subjects. On the right side, the mean distance was 7.22 ± 3.19 mm in edentulous subjects and 7.23 ± 2.69 mm in dentulous subjects (p = 0.978). On the left side, the corresponding values were 7.21 ± 2.81 mm and 7.00 ± 2.52 mm, respectively (p = 0.667), as shown in Table 4.

**Table 4:** A comparison of mean distance between maxillary sinus floor and alveolar ridge according to dentition status. The table summarizes the mean distance (in mm) between the maxillary sinus floor and the alveolar ridge on the right and left sides in dentulous and edentulous subjects. Data are presented as mean ± standard deviation. The mean difference, t-value, and p-value were derived using an independent samples t-test to evaluate statistical significance between groups based on dentition status.

Variables	Status of dentition	N	Mean	Std. Deviation	Mean diff	T value	P value
Position of maxillary sinus right	Edentulous	56	7.2243	3.199	0.0149	0.028	0.978
	Dentulous	64	7.2392	2.695			
Position of maxillary sinus left	Edentulous	56	7.2109	2.8107	0.21	0.432	0.667
	Dentulous	64	7.0006	2.5254			

Chi-square analysis revealed no significant association between gender and the location of maxillary sinus septa on either the right (χ² = 1.56, p = 0.67) or left side (χ² = 2.24, p = 0.523). Similarly, no significant association was observed between gender and the location of the PSAA on the left (χ² = 1.42, p = 0.49) or right side (χ² = 1.42, p = 0.49). The association between gender and dentition status was also not statistically significant (χ² = 0.462, p = 0.265). Furthermore, dentition status did not show any significant association with the location of maxillary sinus septa on the right (χ² = 0.506, p = 0.92) or left side (χ² = 1.66, p = 0.79). Likewise, no significant association was found between dentition status and the location of the PSAA on the right (χ² = 0.86, p = 0.649) or left side (χ² = 2.34, p = 0.3). Overall, the findings indicate that although variations exist in the anatomical location of maxillary sinus septa and the PSAA, these variations are not significantly influenced by gender or dentition status.

Reliability analysis was performed to evaluate the reproducibility and consistency of the CBCT measurements. Cohen's kappa statistics were employed for categorical variables, while the ICC was used for continuous variables. The assessment of sinus septa location demonstrated fair agreement (κ = 0.303; 95% CI: 0.193-0.411). In contrast, the evaluation of PSAA location showed almost perfect agreement (κ = 0.928; 95% CI: 0.858-0.985). Furthermore, the ICC value for maxillary sinus floor distance measurements was 0.777 (95% CI: 0.679-0.853), reflecting good measurement reliability.

## Discussion

The present investigation examined the occurrence of maxillary sinus septa, the anatomical pathway of the PSAA, and their potential relationships with sex and dentition status. The findings indicated that sinus septa were absent in the majority of cases, and when identified, they were most frequently located in the anterior region. This predominance of anterior septa is consistent with earlier observations and may be attributed to variations in sinus pneumatization patterns and developmental processes. From a clinical standpoint, anteriorly positioned septa are of particular importance, as they may increase the likelihood of Schneiderian membrane perforation during sinus-related surgical procedures, especially when evaluated using CBCT imaging [3].

Analysis of the PSAA revealed that the intraosseous course was the most prevalent pattern on both sides, whereas intrasinus and superficial pathways were observed less frequently. These findings are in agreement with previous anatomical and radiographic studies, which describe the artery as commonly embedded within the lateral wall of the maxillary sinus [8,9]. Clinically, the intraosseous location is relevant during lateral window approaches, as inadvertent injury to the vessel may lead to hemorrhage and reduced surgical visibility. In contrast, arteries with an intrasinus course may present a comparatively higher risk because of their proximity to the sinus membrane. Therefore, careful preoperative assessment of vascular anatomy is essential prior to surgical intervention [9,10].

No statistically significant differences were identified between male and female participants with respect to septal distribution, arterial course, or the measured vertical distance between the sinus floor and the alveolar crest. Although males demonstrated slightly higher mean values, these differences were not statistically meaningful, suggesting that sex does not substantially influence these anatomical characteristics [11,12]. These observations are consistent with prior research indicating that, despite variations in craniofacial dimensions, the anatomical relationships within the posterior maxilla remain relatively stable across sexes [11].

Similarly, dentition status did not demonstrate a significant association with septal presence, PSAA configuration, or sinus floor height. While tooth loss is known to contribute to alveolar bone resorption and sinus expansion, the current findings suggest that such changes do not markedly alter the evaluated anatomical parameters [13]. This may reflect individual variability in bone remodeling and sinus pneumatization. Collectively, these results support existing evidence that dentition status alone is not a reliable predictor of anatomical variations in the maxillary sinus or PSAA [14,15]. Consequently, detailed CBCT evaluation remains indispensable for accurate diagnosis and safe surgical planning in the posterior maxillary region [16].

Variations in sinus-root relationships among different populations have been documented in previous literature. Such differences may be influenced by ethnicity, craniofacial morphology, age-related sinus pneumatization, and dentition status. Therefore, population-specific CBCT studies are valuable for improving anatomical understanding and enhancing the safety of surgical procedures in regional populations [17]. Consistent with previous studies, the vertical distance between the alveolar crest and the maxillary sinus floor was greater in the premolar region and relatively reduced in the molar region. This anatomical variation may be attributed to the downward extension of the maxillary sinus in the posterior maxilla. As a result, the molar region requires increased surgical caution during implant placement and sinus augmentation procedures because of the decreased residual bone height and the closer proximity of adjacent vascular structures [18].

The findings of the previous study further emphasize the importance of CBCT as a preferred imaging modality for evaluation of the posterior maxilla. In comparison with conventional radiographic techniques, CBCT provides enhanced visualization of anatomical variations and pathological conditions that may not be readily identifiable on two-dimensional imaging. Such precise radiographic assessment can improve surgical planning, minimize intraoperative complications, and enhance the predictability and success of implant placement and sinus augmentation procedures [19]. No significant associations were identified between demographic variables and the anatomical features evaluated in the study by Gulec et al., suggesting that maxillary sinus anatomy exhibits considerable individual variation. While anatomical variations were frequently detected, their distribution did not appear to be significantly affected by sex or dentition status. Consequently, preoperative CBCT assessment should be performed on an individual basis to ensure accurate surgical planning rather than relying on demographic predictors alone [20].

Limitations of the study

Several limitations should be acknowledged when interpreting the findings of this study. Although a prospective design was employed, the use of CBCT scans originally acquired for diagnostic purposes may have introduced selection bias. The relatively modest sample size (120 participants) may also limit the statistical power of the analysis. In addition, the study population was restricted to a single geographic region (Chhattisgarh), which may reduce the generalizability of the results to populations with differing demographic or ethnic characteristics.

In addition, observer reliability analysis was not comprehensively evaluated. Although all measurements were performed by a single calibrated examiner to maintain consistency, the absence of interobserver reliability assessment remains a methodological limitation. Furthermore, the statistical analysis was primarily limited to descriptive statistics, chi-square tests, and independent samples t-tests. More advanced statistical models and multivariate analyses could provide deeper insight into the potential relationships among anatomical variables and associated factors. Future studies incorporating larger multicenter populations, clinical and surgical correlation, observer reliability assessment, and more comprehensive statistical approaches are recommended to enhance the scientific validity and clinical relevance of the findings.

## Conclusions

This CBCT-based study evaluated the demographic characteristics, dentition status, and maxillary sinus septa distribution among the study population. The findings demonstrated a relatively balanced gender distribution, with a slight predominance of male participants. Dentulous individuals constituted a marginally higher proportion of the sample than edentulous subjects. Maxillary sinus septa were present in a substantial number of cases, indicating that septal anatomy is a common anatomical variation. Septa were observed more frequently on the left side, with the middle region being the most common location, followed by the anterior and posterior regions. These findings emphasize the importance of thorough preoperative CBCT assessment for accurate identification of maxillary sinus septa, which can aid in treatment planning and help minimize surgical complications during sinus augmentation and implant placement procedures.

The PSAA most frequently exhibited an intraosseous course, followed by an intrasinus and superficial course, demonstrating a consistent anatomical pattern bilaterally. Measurements of the distance between the maxillary sinus floor and alveolar ridge showed no statistically significant sex-related differences in the first molar region. These findings highlight the anatomical variability of the maxillary sinus and its associated neurovascular structures, emphasizing the importance of comprehensive preoperative CBCT evaluation before implant placement and sinus augmentation procedures. Accurate identification of sinus septa, assessment of PSAA location, and evaluation of residual alveolar bone height can facilitate surgical planning, reduce intraoperative complications, and contribute to more predictable treatment outcomes in the posterior maxilla.
